# Long Non-Coding RNAs in Multidrug Resistance of Glioblastoma

**DOI:** 10.3390/genes12030455

**Published:** 2021-03-23

**Authors:** Parvaneh Mahinfar, Behzad Baradaran, Sadaf Davoudian, Fatemeh Vahidian, William Chi-Shing Cho, Behzad Mansoori

**Affiliations:** 1 Immunology Research Center, Tabriz University of Medical Sciences, Tabriz 5166/15731, Iran; parvaneh.mahinfar2020@gmail.com (P.M.); baradaranb@tbzmed.ac.ir (B.B.); f_vahidian1370@yahoo.com (F.V.); 2 Humanitas Clinical and Research Center—IRCCS, 20089 Milan, Italy; sadaf.davudian68@gmail.com; 3 Department of Clinical Oncology, Queen Elizabeth Hospital, Hong Kong, China; 4 Department of Cancer and Inflammation Research, Institute for Molecular Medicine, University of Southern Denmark, 5230 Odense, Denmark

**Keywords:** multidrug resistance, glioblastoma, lncRNAs, temozolomide

## Abstract

Glioblastoma, also known as glioblastoma multiforme, is the most aggressive brain tumor in adults. Despite the huge advance in developing novel therapeutic strategies for patients with glioblastoma, the appearance of multidrug resistance (MDR) against the common chemotherapeutic agents, including temozolomide, is considered as one of the important causes for the failure of glioblastoma treatment. On the other hand, recent studies have demonstrated the critical roles of long non-coding RNAs (lncRNAs), particularly in the development of MDR in glioblastoma. Therefore, this article aimed to review lncRNA’s contribution to the regulation of MDR and elucidate the underlying mechanisms in glioblastoma, which will open up new lines of inquiry in the treatment of glioblastoma.

## 1. Introduction

Gliomas are identified as highly prevalent forms of brain malignancies in humans [1]. In accordance with the cellular origin of tumors, they are sub-classified into four categories including astrocytomas, oligodendrogliomas, ependymomas, and mixed tumors [2]. In addition, gliomas are also divided into four grades from the histological and genetic points of view. Low-grade gliomas are called grade I and II tumors and high-grade gliomas are considered grade III and IV [3]. Glioblastoma is known as glioblastoma multiforme (GBM) and is placed in the grade IV astrocytoma category. It is known as the most common and aggressive type of gliomas with an extensively low survival rate after diagnosis [4]. The incidence of GBM ranges from 0.59–5/100,000 persons [5]. With the standard therapeutic regimen, including the maximal surgical resection, chemotherapy, and radiation, the survival values for patients is only 12–14 months [4]. In the case of lack of any therapeutic intervention, this value drops to 6 months [6].

Despite the huge efforts in understanding the underlying mechanisms in the initiation/progression of glioblastoma, its etiology is not fully understood. It is well known that there is a great degree of heterogeneity in different features of glioblastoma such as molecular, cellular, and histological characteristics [7]. Due to this heterogeneity, the tumor recurrence and development of multidrug resistance (MDR) to conventional chemotherapeutics are two common events in patients with glioblastoma [7]. MDR is defined as the capability of cancer cells to remain resistant against a broad spectrum of anti-cancer treatments [8]. Reducing the absorption of the drug following the induction of their release outside of the cells may develop the MDR mechanism [9].

Therefore, recent studies have focused on discovering the molecular mechanisms of MDR in glioblastoma and efficient strategies to reverse it. MDR is developed by alteration in a broad range of cellular events, from variation in drug efflux and metabolism to disruption of apoptosis, cell cycle, and DNA repair and dysregulation of non-coding RNAs [10,11,12,13]. At which, long non coding RNAs (lncRNAs) have recently been described as RNA molecules with a length of more than 200 nucleotides, which do not code into protein [14]. LncRNA naturally does not have functional open reading frames (ORFs) but it maintains the protein-coding genes feature, for instance 5′ cap and alternative splicing. Moreover, many of them have two or more exons and a majority of them have polyA tails (about 60%) [15]. These long RNAs have been demonstrated to play active roles in various biological and physiological processes through controlling gene expression at three levels: the transcriptional, post-transcriptional, and epigenetic levels [16]. LncRNAs are transcribed by RNA polymerase II [17]. An accumulating number of recent studies have reported that lncRNAs are involved in the development of MDR to standard chemotherapeutics in various human malignancies including glioblastoma [18]. Therefore, in this review, we first discussed the critical function of lncRNAs in the glioblastoma, then focused on lncRNA’s roles in the development of MDR in glioblastoma, and the lncRNAs potential for targeting MDR.

## 2. LncRNAs: Amazing Non-Coding RNA Molecules

LncRNAs play substantial roles in the cellular biological process by regulating the expression of various target genes, in either a negative or positive manner [19]. This is achieved by direct interference with gene promoters or modification of chromatin structures [20]. In addition, lncRNAs can interact with specific proteins, and alter their localization or their structure, and hence modulate their activity [20]. LncRNAs are precursors of small RNAs and are involved in the processing of other RNA molecules into small ones [21,22]. More importantly, recent evidence has demonstrated the presence of a sophisticated regulatory network between lncRNAs and miRNAs with critical roles in the regulation of various pathophysiological processes [22]. There are two classifications for lncRNAs according to their genomic position and origin and biological roles and functions.

## 3. Genomic Position and Origin

**Figure 1:**
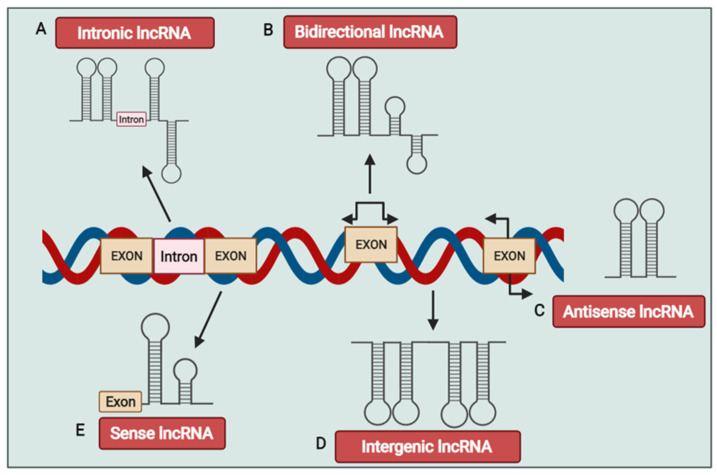
Overview of long non-coding RNA (lncRNA) classification. Based on the genomic position and origin, lncRNAs classified in 5 group including sense, antisense, bidirectional, intronic, and intergenic lncRNAs. Interonic lncRNAs are transcribed in the genomic region between two coding areas (Exons) (**A**). Bidirectional lncRNAs are transcribed from the opposite strand, in the opposite direction (**B**). Antisense lncRNAs are transcribed from the opposite strand of coding exons (**C**). Intergenic lncRNAs are transcribed from the intergenic regions of protein coding genes (**D**). Sense lncRNAs are transcribed from the sense strand of an intronic region with no overlap of exonic sequence (**E**).

lncRNAs are classified into five groups in accordance with their genomic position and origin [23]. These groups are sense, antisense, bidirectional, intronic, and intergenic (Figure 1). Sense lncRNAs are RNA molecules overlapping with one or more exons of specific genes, which have transcription direction to the coding gene. Antisense lncRNAs also overlap with exons of specific coding genes with a transcription direction opposite to the corresponding gene. Bidirectional lncRNAs are located in the proximity of the protein-coding genes, opposite the DNA strand. The fourth group (intronic lncRNAs) is located in the intronic region of coding genes. Finally, the fifth group, the intergenic lncRNAs, originate from a genomic sequence between the two independent coding genes [23]. 

## 4. Biological Roles and Functions

**Figure 2:**
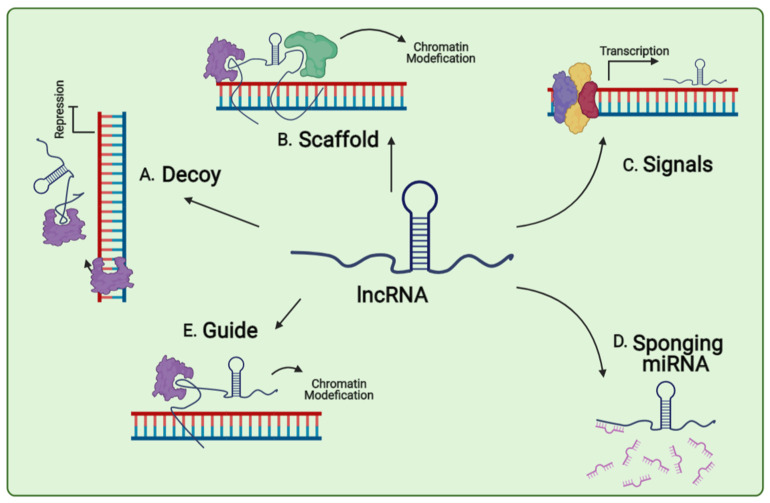
Overview of lncRNAs functions. Decoy lncRNAs can sponge protein factors such as transcription factors and chromatin modifiers (**A**). Scaffold lncRNA is involved in assembly of complex protein complexes (**B**). Signals lncRNAs can serve as molecular signals and can act as markers of functionally significant biological events (**C**). Sponging miRNA lncRNA participate in miRNA inactivation by sponging them (**D**). Guide lncRNAs can be molecular guides by localizing particular ribonucleoprotein complexes to specific chromatin targets (**E**).

lncRNAs are classified into five groups based on their biological roles and functions in the cells, including signals, molecular decoys, molecular guides, and scaffolds. Signal lncRNAs are involved in responding to the various stimuli, in a spatiotemporal manner, so that their expressions reflect the modulation of the gene and can be a marker for major biological events (Figure 2) [24,25]. lncRNAs interact with specific effector proteins that are involved in chromatin modification or gene expression, and later, their biological functions are considered as a molecular decoy (Figure 2) [26]. lncRNAs with a molecular guide direct the ribonucleoproteins to their specific locations on chromatin by binding to them. These lncRNAs exert regulatory effects on gene expression in two manners; on neighboring genes or cis manner, or distantly located genes, trans manner [27]. The fourth group of lncRNAs is considered scaffolds that serve as main platforms for assembling the distinct proteins as a chromatin-modifying complex, hence activating or suppressing the expression of target genes (Figure 2). The last and recently identified group is called ceRNAs, which act as miRNA sponges (Figure 2) [28]. These lncRNAs bind to miRNA through their miRNA-specific binding sites and regulate the miRNA expression and function [28]. Other than miRNA sponging, there are two other hypotheses which describe how lncRNAs can be involved in the regulation of miRNAs. The first hypothesis claims that they may act as precursors of miRNAs generation and the second hypothesis proposes their role in miRNA binding alteration through the direct competition for binding sites [29]. lncRNAs are transcribed in stage-specific and cell type manners with low expression levels in the nucleus [30]. In addition, some lncRNAs are located in the cytoplasm. The subcellular localization of lncRNAs explains the approach to interact with partners and contributes to the regulation of their functional models [31,32]. Among 60,000–100,000 lncRNA genes identified within the human genome, only 200 of them are well-known [33]. Since lncRNAs show cell- and tissue-specific expression patterns, it is suggested that these RNA molecules possess important physiological and biological functions [34]. Therefore, previous studies have reported the involvement of lncRNAs in numerous phases of cell regulation including cell growth and proliferation, differentiation, cell death, genomic imprinting, epigenetic regulation, and alternative splicing, through modulating the chromatin-modifying complexes or DNA regulatory elements at transcriptional and post-transcriptional levels [35]. The versatile nature of lncRNAs is suggested as the possible reason for the diversity of their functions. The capability of RNA molecules for constructing the secondary structures leads to their interaction with multiple highly specific substrates [36]. In addition, non-coding RNAs are highly dynamic transcripts and their levels rapidly alter to ensure the efficient regulation of gene expression [36].

## 5. LncRNAs in Cancer Hallmark

Genetic alterations and aberrant expression patterns in key genes which are related to the balance of cellular proliferation and death are considered the two main reasons for the malignant transformation of cells and cancer development. More importantly, genome-wide association investigations have indicated the occurrence of a majority number (more than 80%) of single nucleotide polymorphisms (SNPs) in non-coding regions of the whole genome, which are transcribed into lncRNAs. Therefore, these long non-coding RNA molecules are revealed to play crucial functions in tumorigenesis [21,24,37,38,39,40,41]. LncRNAs, as a highly heterogeneous group of RNA molecules, are involved in the regulation of gene expression through different mechanisms. LncRNA expression has been correlated to distinguished gene sets with a major impact on cell growth, cell cycle regulation, cell death, cell mobility, survival, pluripotency, and immune response, which contribute to the malignant transformation of normal cells into cancer cells. Not surprisingly, tumor cells showed an aberrant expression pattern for lncRNAs. This dysregulation in the expression levels of lncRNAs in cancer cells has a strong association with the cancer cell potential to initiate the tumor growth and metastasis, and reduce the patient survival rate in a broad range of human malignancies [21,24,37,38,39,40,42]. There are various mechanisms suggested for the regulatory role of lncRNAs in cancer. It is demonstrated that lncRNAs act as oncogenes and/or tumor suppressors in various cancers. Some important examples of oncogenic lncRNAs, which are upregulated in cancers and can trigger cancer progression, include: HOX transcript antisense RNA (HOTAIR), carboxyl-terminal binding protein-antisense (CTBP-AS), antisense non-coding RNA in the INK4 locus (ANRIL), hepatocellular carcinoma up-regulated long non-coding RNA (HULC), HOXA transcript at the distal tip (HOTTIP), breast cancer anti-estrogen resistance 4 (BCAR4), H19, prostate cancer-associated transcript (PCAT)1/5/18, hepatocellular carcinoma up-regulated EZH2-associated long non-coding RNA (HEIH), metastasis associated lung adenocarcinoma transcript 1 (MALAT1), KCNQ1 opposite strand/antisense transcript 1 (KCNQ1OT1), prostate cancer gene expression marker 1 (PCGEM1), long stress-induced non-coding transcripts 5 (LSINCT5), urothelial cancer-associated 1 (UCA1), X-inactive specific transcript (XIST), SWI/SNF complex antagonist associated with prostate cancer 1 (SCHLAP1), taurine upregulated gene 1 (TUG1), and human plasmacytoma variant translocation 1 (PVT1). Tumor suppressor lncRNAs, which are downregulated in cancer cells, and their decreased expression levels lead to malignant transformation, include: GAS5, BGL3, TERRA, DILC, MEG3, DLEU1/2, PTENP1, and NBAT-1 [21,42,43]. The numbers of lncRNAs in the list of oncogenic and tumor suppressor lncRNAs are increasing every year by identifying the novel lncRNAs through profound evaluations and novel techniques, which highlight the undeniable importance of these RNAs in tumorigenesis and cancer therapy. 

## 6. Expression Profile of LncRNA in Glioma and Glioblastoma

Due to the importance of lncRNAs in the gene regulation and critical involvement of various lncRNAs in the pathogenesis of a broad range of human malignancies, an increasing number of recent studies have examined the expression pattern of lncRNAs in gliomas and glioblastoma. For example, in a study by Han et al. [44], authors compared the expression profiles of lncRNA and mRNA in normal brain tissues and glioblastoma. Data revealed 654 up-regulated and 654 down-regulated lncRNAs in glioblastoma brain tissues. In addition, 104 matched lncRNA-mRNA pairs were found for 91 differentially expressed lncRNAs and 84 differentially expressed genes [44]. In another study by Zhang et al. [45], data showed that 129 lncRNAs had differentiated expression levels in glioma and non-tumor brain tissues of 268 clinical specimens. More importantly, they observed a strong association between the expression levels of particular lncRNAs and different histological subtypes and malignancy grades [45]. Murat et al. reported 37 up-regulated and 44 down-regulated lncRNAs in glioma [46,47]. Grzmil et al. evaluated 147 lncRNAs with unique expression levels in 30 glioma samples, as compared with normal brain tissues [47,48]. Li et al. described 398 lncRNAs with distinctive expression levels in glioblastoma and normal brain tissues [49]. Various studies have demonstrated a remarkable correlation between lncRNAs expression levels and glioma malignancy grade [45,50]. Therefore, disruption in the expression levels of lncRNA has a pivotal function in gliomagenesis, and many studies have examined various lncRNAs roles in different aspects of glioblastoma biology such as cell proliferation, apoptosis, angiogenesis, and metastasis. These studies are summarized in Table 1. 

In order to evaluate the genetic profile in tumor tissue, extraction of the tumor is invasive, expensive, and risky for the patients. On the other hand, a small and localized biopsy from tumor tissues might not show a full capture of intratumoral heterogeneity. Therefore, in recent years liquid biopsy has attracted oncologists’ attention as a promising source of biomarkers for diagnostic and prognostic purposes. The biological analytes such as circulating tumor cells (CTCs), circulating cell-free tumor RNA, and tumor-derived exosomes contain circulating lncRNA are found in body fluids such as a serum, CNS (central nervous system) fluid, etc. [51]. Tan et al. showed that HOTAIR expression in serum exosomes and exosome-depleted supernatant of glioblastoma patients was significantly higher than normal control [52]. In another study, Shen et al. measured the six known oncogenic lncRNAs, CRNDE, GAS5, H19, HOTAIR, MALAT1, and TUG1, in serum samples of 106 glioblastoma patients. Among these six lncRNAs, HOTAIR and GAS5 increased expression could serve as reciprocal prognostic predictors of patient’s survival and disease progression [53]. These findings support that evaluating the lncRNA could help to accelerate the diagnosis and prognosis of glioblastoma patients.

## 7. Drug Resistance in Glioblastoma

**Figure 3:**
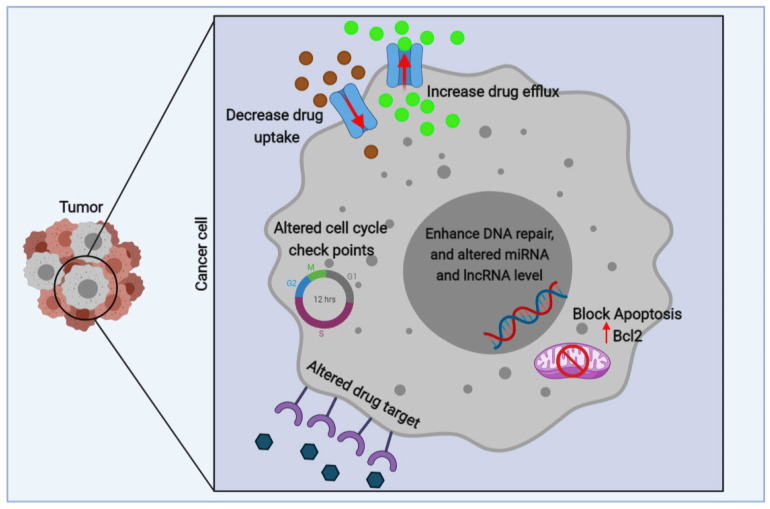
A scheme of the mechanisms involved in multidrug resistance in tumor cells. The mechanisms involved in multidrug resistance (MDR) overexpression include 1. An increase in ATP-binding cassette (ABC) transporters which increases drug efflux, which reduces intracellular drug concentration. 2. Reducing drug uptake by influx transporters. 3. Blocking apoptotic signaling pathways and increase anti-apoptotic (B-cell lymphoma 2) Bcl2. 4. Enhancing DNA repair and increasing adaptability by miRNA and lncRNA regulation. 5. Mutations in drug targets. 6. Aberrant expression of cell cycle checkpoints.

Similar to other cancer types, the development of MDR against conventional chemotherapeutics is one of the most important burdens against the complete and successful treatment of glioblastoma, as well as tumor recurrence following the complement of therapy regimen. Various mechanisms have been identified and studied for the development of MDR in cancer cells, among them, the upregulation of drug transporters, alteration in drug metabolisms, apoptosis suppression, aberrant expression of cell cycle checkpoints, dysregulation of DNA repair machinery, epithelial-to-mesenchymal transition (EMT) cancer stem cells, and the disruption to miRNA and lncRNA expression pattern are considered important ones (Figure 3). Temozolomide is the main chemotherapeutic agent with high efficiency in killing glioblastoma cells. It is reported that temozolomide chemotherapy can increase the patient survival by 2.5 months. However, the main challenge of temozolomide usage is, evolving a “hypermutator” phenotype in glioblastoma cells [100]. Temozolomide mainly leads to mutations in retinoblastoma (RB) and AKT/mammalian target of rapamycin (mTOR) signaling, which plays a critical role in glioblastoma tumor growth and metastasis [101]. Following entrance to the blood circulation, temozolomide undergoes a spontaneous conversion to 5-(3-Methyl-1-triazeno) imidazole-4-carboxamide. This active form of temozolomide is then broken down and generates the methyl diazonium cation. DNA methylation at adenines and guanines, which are mediated by methyl diazonium cation, causes the irreparable DNA damage and cell death [102]. Overexpression of O^6^-methyl-guanine-DNA methyltransferase (MGMT) is considered one of the main mechanisms for the development of MDR against temozolomide. This enzyme catalyzes a reaction, resulting in the removal of the methyl group from the O^6^ of guanine. Methylation in the MGMT promoter, which leads to its downregulation was shown to increase the temozolomide efficacy and prolong the patient survival [103]. It was reported that NEAT1 regulates MGMT in temozolomide resistance [104], NEAT1 was enhanced in TMZ-resistant GBM cells. The presence of temozolomide-resistant glioblastoma cancer stem cells is another substantial factor that contributes to temozolomide MDR [105]. Nevertheless, an accumulating number of recent studies have demonstrated that lncRNAs also mainly contribute to temozolomide MDR, which will be discussed further in the following section.

## 8. LncRNAs in Glioblastoma Drug Resistance

Various studies have investigated the involvement of multiple lncRNAs in the development of MDR or the efficacy of targeting lncRNAs in reversing MDR in glioblastoma. Evaluating the expression pattern of various lncRNAs in temozolomide-resistant glioblastoma tissues revealed that 299 lncRNAs have aberrant expression patterns in glioblastoma tissues in comparison to the normal tissues [106]. In another study, 22 lncRNAs were found with high expression in reversing MDR in glioblastoma [107]. MALAT-1 and H19 are well-studied lncRNAs in developing temozolomide MDR in glioblastoma. Herein, we list some well-known lncRNAs in temozolomide-resistance and their mechanisms of action in glioblastoma (Table 2).

**Figure 4:**
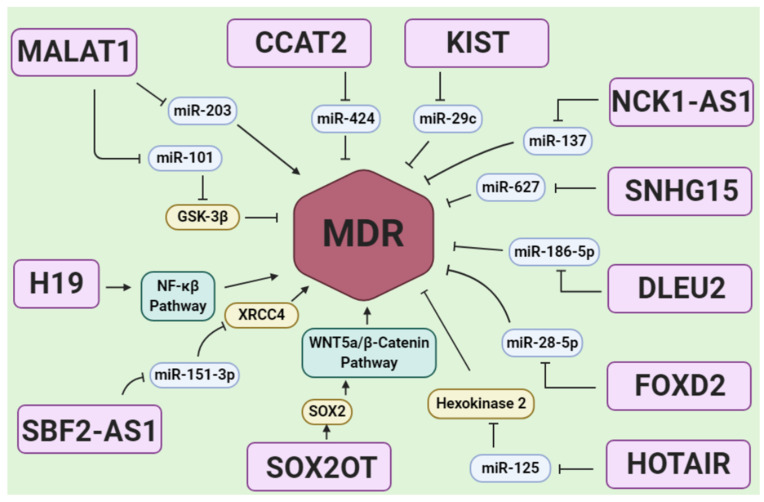
A scheme of lncRNAs in glioblastoma drug resistance via regulating MDR. Most of the lncRNA regulation on MDR is mediated by miRNA sponging in glioblastoma drug resistance. miRNA sponging causes MDR activation or MDR inhibition, and they may regulate via a miRNA direct sponge effect on MDR or sponging the miRNA, which is involved in targeting genes related to MDR regulation such as Sox2, XRCC4, Hexokinase 2, and GSK-3β. In addition, some lncRNAs including H19 and SOX2OT could activate signals such as NF-kB and WNT5a/β-Catenin which can activate MDR.

MALAT-1, also known as nuclear-enriched abundant transcript 2 (NEAT-2), is one of the oncogenic lncRNAs with high expression level in glioblastoma tissues [129]. Its importance in temozolomide-resistance is highly addressed by various studies showing the aberrant expression levels of this lncRNA in resistant glioblastoma cells. Voce et al. [130] showed temozolomide treatment-induced lncRNA MALAT1 in an NF-κB and p53 co-dependent manner in glioblastoma cells, which supported MALAT1 as a target for chemosensitization of glioblastoma [130]. In a study by Chen et al. [108], the upregulation of MALAT-1 was demonstrated in temozolomide-resistant glioblastoma patients’ tissues. Temozolomide-resistant U251 and U87 cells also showed a similar pattern. Investigating the underlying mechanism has revealed that MALAT-1 inhibited miR-203 expression, and hence led to MDR development [108] (Figure 4). Li et al. [109] showed that siRNA-mediated silencing of MALAT-1 in temozolomide-resistant U251 and U87 cells resulted in reversing MDR in these cells. It was found that downregulating MALAT-1 results in significant inhibition of major drug transporters expression levels, such as multidrug resistance protein 1 (MDR1) and multidrug resistance-associated protein 5 (MRP5), as well as modulation of EMT through targeting E-box-binding homeobox 1 (ZEB1), E-cadherin, and zonula occludens-1 (ZO-1) [109]. In another study, it was reported that MALAT-1 knockdown resulted in the upregulation of miR-101 and downregulation of glycogen synthase kinase (GSK)-3β in resistant glioblastoma cells, hence overcoming the temozolomide-MDR in the cells (Figure 4) [110]. Kim et al. indicated that attenuation of MALAT-1 by siRNA significantly decreased the growth, motility, and stemness of glioblastoma cells. Moreover, silencing of MALAT-1 increased the sensitivity of glioblastoma cells to temozolomide [111]. 

H19 is another oncogenic lncRNA which is commonly overexpressed in glioblastoma tissues and cells [131]. H19 was highly upregulated in temozolomide-resistant U251 and M059J glioblastoma cells. H19 downregulation resulted in notable MDR reversal in resistant cells through inhibiting the expression of EMT markers and suppressing Wnt/β-catenin signaling [112]. Jiang et al. reported the presence of three distinct variants for H19 [113]. In temozolomide-resistant glioblastoma tissues, the expression levels of variant 1 were higher in comparison to the other two variants. The possible underlying mechanisms for H19-mediated MDR in glioblastoma are the overexpression of MDR, MRP, and ATP-binding cassette superfamily G member 2 (ABCG2) [113]. Activation of the NF-κB signaling pathway is considered as another mechanism for H19-mediated MDR in glioblastoma (Figure 4) [114].

Small nucleolar RNA host gene 12 (SNHG12) is an oncogenic lncRNA, which is aberrantly expressed in temozolomide-resistant glioblastoma cells and tissues. Lu et al. [115] reported that SNHG12 acts as a sponge for miR-129-5p, therefore leading to elevated expression of MAPK1 and E2F7, which in consequence promotes cell proliferation, suppresses cell apoptosis, and enhances the development of temozolomide resistance in glioblastoma cells. In addition, SNHG12 knockdown led to chemosensitization in resistant cells [115]. Another overexpressed lncRNA in temozolomide-resistant glioblastoma cells is SBF2 antisense RNA 1 (SBF2-AS1) (Figure 4) [116]. It was demonstrated that SBF2-AS1 serves as a ceRNA for miR-151a-3p and by targeting X-ray repairs the cross-complementing 4 (XRCC4), which is a key component of DNA repair machinery, increased the glioblastoma cells’ ability to repair the DNA damages. For this reason, SBF2-AS1 enhanced the temozolomide-MDR in glioblastoma cells through increasing the DNA repair capacity [116]. lncRNA LINC00461 was also indicated as ceRNA for miR-216a [117]. This oncogenic lncRNA promoted cell proliferation, migration, and invasion in glioblastoma, as well as temozolomide resistance through targeting the miR-216a and aquaporin 4 pathway. Thus, silencing LINC00461 led to remarkable chemosensitization in glioblastoma cells [117]. CCAT2 by sponging miR-424 [118], XIST by targeting miR-29c [119], NCK1-AS1 by targeting miR-137 [120], SNHG15 by targeting miR-627 [106], DLEU2 by targeting miR-186-5p [121], and FOXD2 by sponging miR-98-5p [122] play a major role in the promoting cell proliferation and invasion and developing MDR against temozolomide in glioblastoma cells (Figure 4). 

In addition to miRNAs, lncRNAs were reported to increase the chemoresistance in glioblastoma cells by targeting various proteins. ADAM metallopeptidase thrombospondin type 1 motif 9 antisense RNA 2 (ADAMTS9-AS2), with an upregulated pattern in temozolomide-resistant glioblastoma cells, was reported to promote the resistant phenotype. Upregulating the fused in sarcoma (FUS), which is an RNA-binding protein associated with ADAMTS9-AS2 and E3 ubiquitin-protein ligase MDM2 [132]. LINC01198 promoted the drug resistance in glioblastoma cells through upregulating neural precursor cells and developmentally downregulated 4, E3 ubiquitin-protein ligase (NEDD4-1) and phosphatase and tensin homolog (PTEN) [123]. NEAT1 and FoxD2-AS1 hypermethylated of the promoter region of MGMT, hence mediate temozolomide resistance [133,134]. LncRNA MIR155HG induced temozolomide resistance in glioma cells through targeting and activating the Wnt/β-catenin pathway [124]. LncRNA SOX2OT was involved in the development of MDR against temozolomide in glioblastoma cells through upregulating SOX2 expression, which activated the Wnt5a/β-catenin signaling pathway (Figure 4) [125]. EPIC1 lncRNA plays a critical role in the temozolomide resistance by targeting Cdc20 [16]. 

In addition to various lncRNAs with MDR promoting roles, some lncRNAs inhibit MDR in glioblastoma. For example, Zhang et al. [126] described the critical role of the inhibitory function of HOTAIR lncRNA in developing MDR against temozolomide in glioblastoma cells. Resistant glioblastoma cells showed decreased expression levels of HOTAIR. Overexpressing this lncRNA led to elevated expression levels of Hexokinase 2 by targeting miR-125, hence impeding the cell growth and enhancing the temozolomide-induced apoptosis (Figure 4) [126]. Xu et al. [127] showed that overexpression of lncRNA AC003092.1 overcame temozolomide resistance by modulation of miR-195/TFPI-2 signaling in glioblastoma. TUSC7 is another lncRNA that inhibited temozolomide resistance by targeting miR-10a in glioblastoma cells [128].

## 9. Conclusions

The present review has demonstrated the major involvement of various lncRNAs in the development of MDR in glioblastoma. Targeting MDR transporters, modulating apoptosis, targeting DNA repair machinery, controlling cancer stem cells, regulating EMT, and crosstalk with major pro-oncogenic signaling pathways are among the most important mechanisms in miRNAs-mediated MDR in glioblastoma. Therefore, targeting lncRNAs seem to be an appropriate strategy for combating treatment obstacles in glioblastoma. 

On the other hand, there is still limited knowledge about the sophisticated interactions and effects between lncRNAs and genome, hence the lncRNAs implication for overcoming the drug resistance in glioblastoma needs more in-depth research. In addition, as mentioned in some studies, silencing the particular lncRNAs may be an effective strategy to reverse the drug resistance in glioblastoma. Therefore, targeting and delivering strategies such as using nanoparticles and extracellular vesicles in carrying lncRNA (exosomes) for lncRNAs is another issue that needs to be addressed urgently.

In addition to treatment, the gene expression analysis and reporting the differential gene expression profile of glioblastoma and/or drug resistance glioblastoma samples compared to normal tissues can help to manage both drug-sensitive and drug-resistant forms of this disease.

## Figures and Tables

**Table 1:** LncRNA status in glioma and glioblastoma.

lncRNA	Full Name	Expression Pattern	Targets	Major Finding	Ref.
MALAT1	Metastasis-associated lung adenocarcinoma transcript 1	Upregulated	Wnt signaling	Promotes cell migration, but not proliferation, in glioblastoma cell lines LN-229, LN-18, and LN-428.	[54]
		Downregulated	Erk/MAPK signaling MMP2	Knockdown of MALAT1 promotes cell proliferation and invasion, whereas overexpression of MALAT1 induces reductions in cell proliferation and invasion in U87 and U251 cells and tumorigenicity in both subcutaneous and intracranial human glioma xenograft models.	[55]
		Downregulated	miR-155 FBXW7	Suppression of cell proliferation in U87 and SHG139 cells.	[56]
		Upregulated	Rap1B miR-101	Promotes proliferation and suppresses apoptosis.	[57]
		Upregulated	miR-101 STMN1 RAB5A ATG4D	Activates autophagy and promotes cell proliferation.	[58]
		Upregulated	miR-129 SOX2	Promotes glioma tumorigenesis.	[59]
HOTAIR	Hox transcript antisense intergenic RNA	Upregulated	PRC2	Promotes cell cycle.	[60]
		Upregulated	miR-326 FGF1	Knockdown of HOTAIR was found to exert a suppressive function on cellular proliferation in vitro and in vivo.	[61]
		Upregulated	miR-148b-3p USF1	Knockdown of HOTAIR can also increase the permeability of the blood-tumor barrier (BTB) in glioma microvascular endothelial cells facilitating the delivery of antineoplastic drugs.	[62]
FOXM1-AS	Forkhead box M1 lncRNA	Upregulated	ALKBH5 FOXM1	Promotes tumorigenesis through the FOXM1 axis in vitro and in a mouse intracranial xenograft model.	[63]
HOTTIP	HOXA distal transcript antisense RNA	Downregulated	BRE	Inhibits cell proliferation and cell cycle progression, and it promotes apoptosis.	[64]
		Upregulated	HIF-1α miR-101 ZEB1	In glioma cells treated by hypoxia, HOTTIP is significantly upregulated and associated with the epithelial-mesenchymal transition (EMT) process and metastasis.	[65]
HOXA11-AS	Homeobox A11-AS	Upregulated	HOXA	Promotes cell proliferation by the regulation of cell cycle progression in vitro and in vivo.	[66]
		Upregulated	miR-140-5p	Promotes the glioma tumorigenesis.	[67]
		Upregulated	miR-214-3p EZH2	Promotes growth, migration, and invasion of glioma cells.	[68]
		Upregulated	miR-124-3p	Promotes malignant progression of glioma.	[69]
ECONEXIN	LINC00461	Downregulated	miR-411-5p TOP2A	Decreased cell proliferation.	[70]
GAS5	Growth Arrest-Specific 5	Downregulated	Bm Plexin C1 miR-222	Inhibits cell proliferation. Gas5 knockdown suppresses glioma growth and prolongs the survival of tumor-bearing nude mice in vivo.	[71]
		Downregulated	miR-196a-5p FOXO1	Inhibits glioma cell proliferation, migration, and invasion.	[72]
H19	-	Upregulated	-	Promotes invasion, angiogenesis, and stemness of glioblastoma cells.	[73]
		Upregulated	CD133 NANOG Oct4 Sox2	Increased cellular proliferation and suppressed apoptosis.	[74]
		Upregulated	miR-675 CDK6 Cadherin	Promote glioma cell invasion.	[61,75]
		Upregulated	miR-29a	Regulates glioma angiogenesis and the biological behavior of glioma-associated endothelial cells.	[76]
		Upregulated	miR-140 iASPP	Modulates glioma growth.	[77]
		Upregulated	miR-152	Promotes proliferation and invasion in human glioma cells.	[78]
XIST	X-inactive specific transcript	Upregulated	miR-152	Knockdown exerts tumor-suppressive functions in human glioblastoma stem cells.	[79]
		Upregulated	miR-137	Knockdown of long non-coding RNA XIST increases blood-tumor barrier permeability and inhibits glioma angiogenesis.	[67]
		Upregulated	miR-137	Exerts oncogenic functions.	[80]
		Upregulated	miR-429	Promotes glioma tumorigenicity and angiogenesis.	[81]
CRNDE	-	Upregulated	mTOR signaling	Promotes glioma cell growth and invasion.	[82]
		Upregulated	miR-186	Affects the malignant biological characteristics of human glioma stem cells.	[71]
		Upregulated	miR-384 PIWIL4 STAT3	Promotes malignant progression of glioma.	[83]
		Upregulated	miR-136-5p Bcl-2 Wnt2	Acts as a ceRNA and promotes glioma malignancy.	[84]
NEAT1	Nuclear-enriched abundant transcript 1	Upregulated	ICAT GSK3B Axin2 EZH2	Promotes glioma cell growth and invasion.	[85]
		Upregulated	miR-449b-5p c-Met	Promotes glioma pathogenesis.	[86]
		Upregulated	let-7e	Knockdown of NEAT1 restrains the malignant progression of glioma stem cells.	[87]
		Upregulated	miR-107 CDK6	Promotes glioma stem-like properties.	[88]
		Upregulated	SOX2 miR-132	Promotes glioma cell migration and invasion.	[89]
		Upregulated	miR-181d-5p ZO-1 occludin claudin-5	Regulates permeability of the blood-tumor barrier.	[90]
HCP5	Histocompatibility leukocyte antigen (HLA) complex P5	Upregulated	microRNA-139 RUNX1	Promotes the malignant behaviour of glioma cells.	[91]
HIF1A-AS2	Hypoxia-inducible factor 1 alpha-antisense RNA 2	Upregulated	-	Controls cellular fate and the molecular landscape of mesenchymal (Glioma stem cells) GSCs maintain the function of mesenchymal GSCs in tumorigenicity and contribute to GSCs’ speciation and adaptation to hypoxic stress.	[92]
RAMP2-AS1		Downregulated	DHC10 NOTCH3 HES1	Cell growth suppression.	[18]
linc-POU3F3	Long intergenic noncoding RNA POU3F3	Upregulated	POU3F3	Promotes cell viability and proliferation.	[93]
CASC2	Cancer susceptibility candidate 2	Downregulated	miR-21	Inhibition of glioma growth, migration, and invasion and promotion of cell apoptosis.	[94]
HMMR-AS1	-	Upregulated	c-Myc	Promotes cell migration, invasion, and mesenchymal phenotypes.	[95]
MEG3	Maternally expressed gene 3	Downregulated	miR-96-5p MTSS1	Suppresses the growth of glioma cells.	[96]
FLVCR1-AS1	Feline leukemia virus subgroup C cellular Receptor 1 Antisense RNA 1	Upregulated	miR-30b-3p	Promotes glioma cell proliferation and invasion.	[97]
MIR22HG	MIR22 host gene	Upregulated	Wnt/β-catenin signalling	Increase cell proliferation, invasion, and in vivo tumor growth.	[98]
linc00645	Long intergenic non-protein coding RNA 645	Upregulated	miR-205-3p	Modulates (Transforming growth factor beta) TGF-β-induced glioma cell migration and invasion.	[99]

**Table 2:** lncRNAs in drug resistance of glioblastoma.

lncRNAs	Expression Pattern in Glioblastoma	Roles in Drug Resistance	Mechanism of Action	Ref.
MALAT1	Upregulated	Temozolomide treatment-induced lncRNA MALAT1. MALAT1 was a target for chemosensitization of glioblastoma.	It was dependent on NF-κB and p53.	[108]
MALAT1	Upregulated	MALAT-1 was upregulated in temozolomide-resistant glioblastoma patients’ tissues.	MALAT-1 inhibited miR-203 expression, hence leading to MDR development.	[108]
MALAT1	Upregulated	siRNA mediated silencing of MALAT-1 in temozolomide-resistant cells reversed MDR.	Downregulating MALAT-1 resulted in the significant inhibition of the expression levels of major drug transporters and modulation of EMT.	[109]
MALAT1	Upregulated	MALAT1 overcame the temozolomide-MDR in cells.	MALAT-1 knockdown resulted in the upregulation of miR-101 and downregulation of glycogen synthase kinase.	[110]
MALAT1	Upregulated	The silencing of MALAT-1 increased the sensitivity of glioblastoma cells to temozolomide.	siRNA significantly decreased the growth, motility, and stemness of glioblastoma cells.	[111]
H19	Upregulated	H19 downregulation resulted in significant reversing of MDR in resistant cells.	H19 downregulation inhibits the expression of EMT markers and suppresses Wnt/β-catenin signaling.	[112]
H19	Upregulated	H19 was upregulated in temozolomide-resistant glioblastoma cells.	Downregulating H19 significantly suppresses the expression levels of major drug transporters.	[113]
H19	Upregulated	H19 was overexpressed in resistant cells.	Activation of the NF-κB signaling pathway was a mechanism for H19-mediated MDR in glioblastoma.	[114]
SNHG12	Upregulated	SNHG12 promoted the development of temozolomide resistance in glioblastoma cells. SNHG12 knockdown led to chemosensitization in resistant cells.	SNHG12 acted as a sponge for miR-129-5p. It increased the expression levels of MAPK1 and E2F7, led to upregulation of MAPK1 and E2F7, hence promoted cell proliferation and suppressing apoptosis.	[115]
SBF2-AS1	Upregulated	SBF2-AS1 enhanced temozolomide-MDR in glioblastoma cells.	It increased DNA repair capacity. SBF2-AS1 serves as a ceRNA for miR-151a-3p.	[116]
LINC00461	Upregulated	It promoted cell proliferation, migration, invasion, in glioblastoma, as well as temozolomide resistance.	LINC00461 was a ceRNA for miR-216a.	[117]
CCAT2	Upregulated	It promoted cell proliferation and invasion and developed MDR against temozolomide in glioblastoma cells.	Sponged miR-424.	[118]
XIST	Upregulated	It promoted cell proliferation and invasion and developed MDR against temozolomide in glioblastoma cells.	Targeted miR-29c.	[119]
NCK1-AS1	Upregulated	It promoted cell proliferation and invasion and developed MDR against temozolomide in glioblastoma cells.	Targeted miR-137.	[120]
SNHG15	Upregulated	It promoted cell proliferation and invasion and developed MDR against temozolomide in glioblastoma cells.	Targeted miR-627.	[106]
DLEU2	Upregulated	It promoted cell proliferation and invasion and developed MDR against temozolomide in glioblastoma cells.	Targeted miR-186-5p.	[121]
FOXD2	Upregulated	It promoted cell proliferation and invasion and developed MDR against temozolomide in glioblastoma cells.	Sponged miR-98-5p.	[122]
LINC01198	Upregulated	It promoted drug resistance in glioblastoma cells.	Upregulated NEDD4-1 and downregulated PTEN.	[123]
MIR155HG	Upregulated	MIR155HG-induced temozolomide resistance.	Targeted and activated the Wnt/β-catenin pathway.	[124]
SOX2OT	Upregulated	SOX2OT is involved in the development of MDR against temozolomide in glioblastoma cells.	Upregulated SOX2 expression, which activated the Wnt5a/β-catenin signaling pathway.	[125]
EPIC1	Upregulated	EPIC1 plays a critical role in temozolomide resistance.	It targeted Cdc20.	[16]
HOTAIR	Downregulated	It had an inhibitory role in developing MDR against temozolomide.	Increased expression levels of Hexokinase 2 by targeting miR-125.	[126]
AC003092.1	Downregulated	Overexpression of lncRNA AC003092.1 overcame temozolomide resistance.	Modulated miR-195/TFPI-2 signaling.	[127]
TUSC7	Downregulated	TUSC7 is inhibited temozolomide resistance.	Targeted miR-10a in glioblastoma cells.	[128]

## Data Availability

Data sharing not applicable.
